# Reduced order modelling and experimental validation of a MEMS gyroscope test-structure exhibiting 1:2 internal resonance

**DOI:** 10.1038/s41598-021-95793-y

**Published:** 2021-08-12

**Authors:** Giorgio Gobat, Valentina Zega, Patrick Fedeli, Luca Guerinoni, Cyril Touzé, Attilio Frangi

**Affiliations:** 1 Politecnico di Milano, Civil and Environmental Engineering, Milan, Italy; 2 Analog and MEMS Group, STMicroelectronics, Cornaredo, Italy; 3 IMSIA, ENSTA Paris, Institut Polytechnique de Paris, Palaiseau, France

**Keywords:** Applied mathematics, Computational science, Mechanical engineering

## Abstract

Micro-Electro-Mechanical Systems revolutionized the consumer market for their small dimensions, high performances and low costs. In recent years, the evolution of the Internet of Things is posing new challenges to MEMS designers that have to deal with complex multiphysics systems experiencing highly nonlinear dynamic responses. To be able to simulate a priori and in real-time the behavior of such systems it is thus becoming mandatory to understand the sources of nonlinearities and avoid them when harmful or exploit them for the design of innovative devices. In this work, we present the first numerical tool able to estimate a priori and in real-time the complex nonlinear responses of MEMS devices without resorting to simplified theories. Moreover, the proposed tool predicts different working conditions without the need of ad-hoc calibration procedures. It consists in a nonlinear Model Order Reduction Technique based on the Implicit Static Condensation that allows to condense the high fidelity FEM models into few degrees of freedom, thus greatly speeding-up the solution phase and improving the design process of MEMS devices. In particular, the 1:2 internal resonance experienced in a MEMS gyroscope test-structure fabricated with a commercial process is numerically investigated and an excellent agreement with experiments is found.

## Introduction

The spread of Micro-Electro-Mechanical Systems (MEMS) in the consumer world triggered a revolution in gaming, mobile phones and navigation. Similarly, in the near future, the evolution of the Internet of Things in its different declinations will require new generations of sensors and actuators with improved performances, smaller dimensions and innovative working principles. As a consequence, MEMS designers more and more frequently will have to deal with complex mechanical structures exhibiting nonlinear dynamic behaviors^1,2^.

Among others, MEMS gyroscopes represent a meaningful example of such a trend. They are electro-mechanical systems able to measure the angular rate by exploiting the Coriolis force. To guarantee a correct functioning, where at least two modes are coupled through the Coriolis force, and to satisfy the strict requirements on the footprint, the mechanical structure is usually very complex, i.e. made by folded springs and rigid masses. Nonlinear phenomena often arise^3–8^ but are difficult to decipher.

Among the wide variety of nonlinear phenomena arising in MEMS devices, internal resonance, i.e. when two or more modes get nonlinearly coupled and exchange energy, is attracting increasing interest for its potential benefits on the performances of MEMS devices^2,9–12^. It has been demonstrated that thanks to internal resonance it is indeed possible (i) to stabilize the oscillation frequency of non-linear self-sustaining micromechanical resonators^13^, (ii) to redistribute and store mechanical energy among vibrational modes and coherently transfer it back to the principal one when the external excitation is off^14^ and (iii) to tune the quality factor *Q* of the driven mode over a wide range^15,16^. Moreover, internal resonance has been recently employed in MEMS gyroscopes as a new and very promising detection technique of angular rate signals^17^ and to design innovative MEMS bandpass filters^18^.

In view of its high potentiality for the design of innovative and high-end MEMS devices, internal resonance has been analyzed theoretically^19^ and experimentally verified on a variety of simple MEMS structures^20–24^, ranging from arch resonators^25–27^ to micro-mirrors^28^. In most cases, the coefficients of Reduced Order Models (ROMs) are obtained from simplified electro-structural theories^26,29^ or are calibrated on experimental data^23^. Despite the great interest of the topic, a general a priori simulation tool that could predict in real-time the nonlinear dynamic behavior of complex MEMS structures like e.g. gyroscopes under different actuation conditions, is still missing. Such a tool would also dramatically improve the design process and pave the way to a new class of sensors/actuators experiencing complex nonlinear dynamic phenomena.

Numerical methods able to simulate the Full Order Model (FOM) have been proposed as a generalization of simplified appoaches^30^, but their computational cost remains a major issue especially if complex MEMS structures are considered. Dedicated Harmonic Balance techniques or shooting procedures are indeed overwhelmingly complex and time consuming^31,32^.

As a consequence, the focus has been set on the generation of nonlinear ROMs starting e.g. from large FEM models that might reshape the governing equations into a nonlinear, dynamical system featuring a much lower dimensionality, yet able to capture the physical features of the problem^29,33–35^. The Stiffness Evaluation Procedure (STEP) in its various variants^33^ assumes a trial subspace spanned by a set of linear modes which however must also include all the coupled high-frequency modes that are often difficult to identify^36,37^. The Proper Orthogonal Decomposition (POD)^38^ is also based on a linear trial space but this is generated from a set of FOM snapshots employing Singular Value Decomposition, thus allowing to identify all the relevant contributions automatically. A different approach is taken by the implicit condensation and expansion (ICE) method^39–41^ which defines a small set of master modes and assumes a quasi-static coupling with the high frequency contributions (slave modes). Also modal derivatives (MD)^42–44^ have been introduced with the aim of accounting for the amplitude dependence of modes. ICE and MD are indeed very accurate when a slow/fast separation between the frequencies of the master and slave modes exist^45,46^. Recently, Nonlinear Normal Modes (NNMs) have received considerable attention as a technique for generating ROMs. Initially defined as a vibration in unison of the system^47–49^, they have been later extended by the notion of invariant manifold^50^ and of spectral submanifold (SSM)^51,52^. However, only very recently efficient approaches have been proposed for the computation of invariant manifolds for large FEM models^53,54^, but applications have been limited so far to mechanical structures with geometrical nonlinearities and no multiphysics coupling.

In this work, we elaborate on the Implicit Condensation approach based on static modal loadings recently tailored by the authors for simple MEMS structures^41^. In particular, the ICE applies to structures which undergo transformations which are no-longer infinitesimal, but still moderate. The approach has been verified on a double-ended tuning fork resonator experiencing both geometric, electrostatic and damping nonlinearities^55^, and represents a fast a priori multiphysics simulation tool able to reproduce the nonlinear dynamics caused by the interaction of two modes of a complex MEMS gyroscope test-structure without the need of calibration procedures. To the authors best knowledge this represents the first fast numerical predictive tool able to simulate a priori the internal resonance phenomenon including bifurcations of the periodic response in a complex structure and in general, the nonlinear dynamic behavior of MEMS devices. Numerical results are compared with experimental data and an excellent agreement is achieved for different actuation conditions, thus proving the versatility and the predictivity of the proposed tool.

## Results

### MEMS gyroscope test-structure

**Figure 1:**
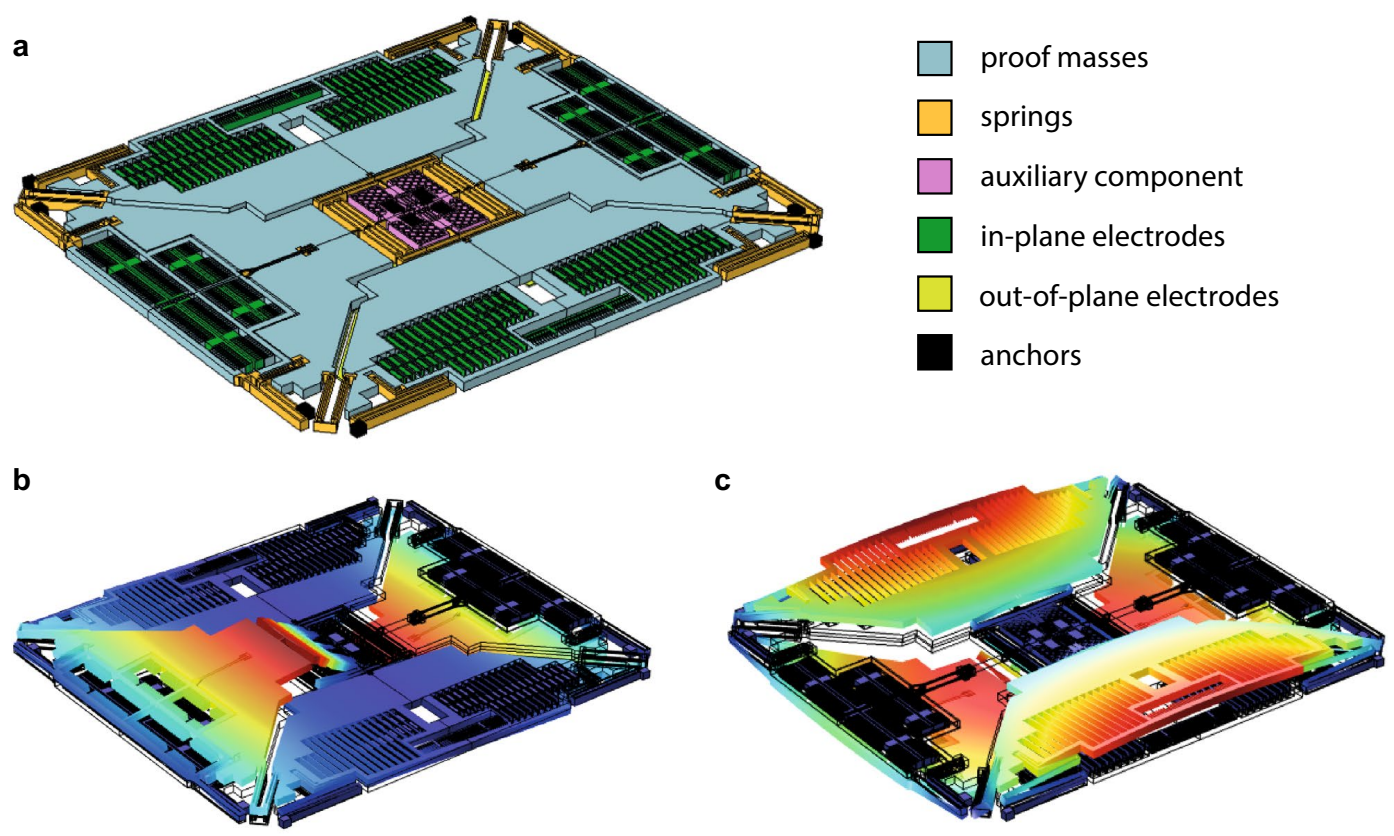
(**a**) Schematic view of the MEMS gyroscope test-structure. (**b**) Roll (*f*_1_ = 22,522 Hz) and (**c**) spurious roll (*f*_2_ = 43,386 Hz) modes. The contour plot of the displacement field is shown in color.

A schematic view of the MEMS gyroscope test-structure employed in this work is reported in Fig. 1a, close-up views and geometrical dimensions are also reported in the Supplementary Information for the sake of clarity. The mechanical structure is constituted by four masses and several folded springs that provide the suspension of the device and the coupling of the masses with a central auxiliary component. The gyroscope test-structure is fabricated through the Thelma process of STMicroelectronics in polysilicon (*E* = 167 GPa, υ = 0.22, ρ = 2330 kg/m^3^) and has an overall footprint of 1.5 mm × 1.3 mm × 24 µm. Comb fingers and parallel plate electrodes allow for the in-plane actuation/readout, while electrodes located on the substrate are employed for the out-of-plane actuation/readout. In Fig. 1b,c, two modes of the MEMS gyroscope test-structure are reported: they will be referred to in the following as roll mode (Fig. 1b) and spurious roll mode (Fig. 1c). Their natural frequencies are computed through a FEM modal analysis and read *f*_1_ = 22,522 Hz and *f*_2_ = 43,386 Hz, respectively.

1:2 internal resonance between the two modes can be triggered by driving the roll mode through the electrodes on the substrate. The two linear natural frequencies have an initial ratio of 1.926, which evolves to almost exactly 2 as the applied electrostatic bias increases and due to the electrostatic nonlinearities given by the parallel-plate electrostatic scheme^55^.

### ROM based on implicit static condensation

A numerical FOM made by discretizing the geometry of the MEMS gyroscopes test-structure with quadratic pentahedrons, properly distributed so as to have at least two elements in the spring thickness, consists of around 2.5 millions of degrees of freedom which become 17 millions if electro-mechanical coupling is considered. This makes any direct numerical simulation computationally unaffordable especially if complex nonlinear dynamic phenomena such as the 1:2 internal resonance are investigated. As an example, we estimate that the simulation of the nonlinear dynamic response of the MEMS quad-mass structure through a fully coupled time domain analyses in COMSOL Multiphysics v.5.6, would require approximatively one year on a standard workstation (AMD Ryzen 9 5950X, 16 Cores, 128 Gb RAM).

The implicit static condensation method validated by the authors on simple structures^41,55^ is here applied to reduce the system to two degrees of freedom (i.e. the amplitudes of the two master roll and spurious-roll modes), thus dramatically reducing the computational effort without losing accuracy and physical meaning (see “Methods” section).

By considering a constant Direct Current (DC) voltage *V*_*DC*_ on the MEMS gyroscope test-structure proof masses and an Alternate Current (AC) signal $V_{AC} \ll V_{DC}$ at an angular frequency $\omega$ close to the one of the roll mode on the electrodes on the substrate, the resulting nonlinear system describing the dynamics of the device reads:

1 $$\ddot{q}_{1} + \frac{{\omega_{01} }}{{Q_{1} }}\dot{q}_{1} + \beta_{1} (q_{1} ,\;q_{2} ) - \check{F}_{{e1_{1} }} (q_{1} ,\;q_{2} )\epsilon_{0} V_{DC}^{2} = 2\epsilon_{0} V_{DC} V_{AC} \check{F}_{{e2_{1} }} (q_{1} ,\;q_{2} )\sin \omega t,$$

2 $$\ddot{q}_{2} + \frac{{\omega_{02} }}{{Q_{2} }}\dot{q}_{2} + \beta_{2} (q_{1} ,\;q_{2} ) - \check{F}_{{e1_{2} }} (q_{1} ,\;q_{2} )\epsilon_{0} V_{DC}^{2} = 0,$$

where $\epsilon_{0}$ is the vacuum permittivity, $q_{i}$ is the modal coordinate, $Q_{i}$ is the quality factor, $\omega_{0i} = 2\pi f_{i}$ is the natural pulsation, $\beta_{i} (q_{1} ,\;q_{2} )$ is the nonlinear mechanical force and $\check{F}_{{e1_{i} }} (q_{1} ,\;q_{2} )$ is a time independent nonlinear electrostatic force for the *i*-th mode, with *i* = 1 for the roll mode and *i* = 2 for the spurious roll mode. $\check{F}_{{e2_{1} }} (q_{1} ,\;q_{2} )$ is the time dependent nonlinear electrostatic force that acts on the driven roll mode. In the following, $Q_{1} = 2400$ and $Q_{2} = 3480$ according to the simplified numerical tool^56^ proposed by the authors to compute fluid damping in MEMS resonant structures working in low pressure conditions such as in this case. Note that a nonlinear quality factor can in principle be also considered in the case of very large displacements of the proof mass with respect to the air gap between it and the fixed electrodes^55^.

**Figure 2:**
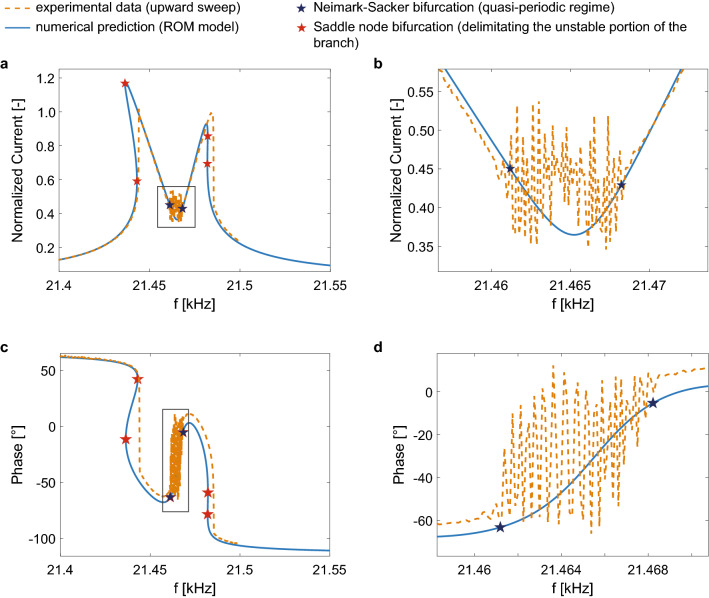
Frequency response of the MEMS gyroscope test-structure for a V_AC_ = 3.16 mV and a V_DC_ = 4.28 V in terms of (**a**) amplitude and (**c**) phase. Close-up views of the quasi-periodic region in the frequency response in terms of amplitude and phase are shown in (**b**)–(**d**). Numerical predictions are plotted with continuous blue lines, and experimental data with orange dashed lines. Stars mark theoretical Neimark–Sacker (dark blue) and Saddle–Node (red) bifurcations that delimit the quasi-periodic regime region and the unstable paths of the frequency response, respectively.

For the sake of simplicity, we approximate $\beta_{i} (q_{1} ,\;q_{2} )$, $\check{F}_{{e1_{i} }} (q_{1} ,\;q_{2} )$ and $\check{F}_{{e2_{1} }} (q_{1} ,\;q_{2} )$ with a complete third order polynomial whose coefficients are reported in the Supplementary Information. Once the voltage levels are fixed, Eqs. (1) and (2) are solved through numerical continuation, using the package MANLAB^57^ that implements a combination of Harmonic Balance (HB) with an asymptotic numerical method (ANM) for path-following. The nonlinear frequency response of the roll mode in terms of amplitude and phase is reported in continuous light blue line in Fig. 2 for a V_DC_ = 4.28 V and a V_AC_ = 3.16 mV. For this actuation condition, the model correctly reproduces the activated 1:2 internal resonance as demonstrated by the characteristic shape of the frequency response made with two peaks and by the presence of a quasi-periodic/chaotic region (see green path in Fig. 2a of Supplementary Information) delimited by Neimark–Sacker bifurcations (dark blue stars in Fig. 2) in the central region of the spectrum^58^. Red stars represent the Saddle–Node bifurcations predicted by the ROM model and delimit the unstable part of the solution branch (see red path in Fig. 2a of Supplementary Information). To further validate the adequacy of the proposed ROM, in the Supplementary Information we report the comparison between the curves obtained through the full ROM here proposed and the ones analytically derived through the Multiple Scale Method^58^ from a simplified ROM based on the coefficients numerically extracted through the Implicit Condensation Method.

In Fig. 2, numerical curves in terms of displacements obtained by integrating Eqs. (1) and (2) are converted in terms of current as detailed in Zega et al.^55^ to simplify the comparison with experimental data.

### Experimental results

In order to validate the proposed simulation tool, the experimental frequency response of the MEMS gyroscope test-structure is measured in the same actuation condition previously considered for the theoretical model: V_DC_ = 4.28 V and V_AC_ = 3.16 mV. Experimental curves are reported in orange dashed lines in Fig. 2 and well agree with the numerical predictions. Note that the jumps of the experimental upward frequency sweep shown in Fig. 2a,c are in a satisfactory agreement with the Saddle–Node bifurcations (red stars) predicted by the models (see Fig. 2a of Supplementary Information). Moreover, in the close-up views of Fig. 2b,d, it is evident that the Neimark–Sacker bifurcations predicted by the ROM correctly delimit the experimental quasi-periodic region, thus further proving the accuracy of the proposed a priori simulation tool.

**Figure 3:**
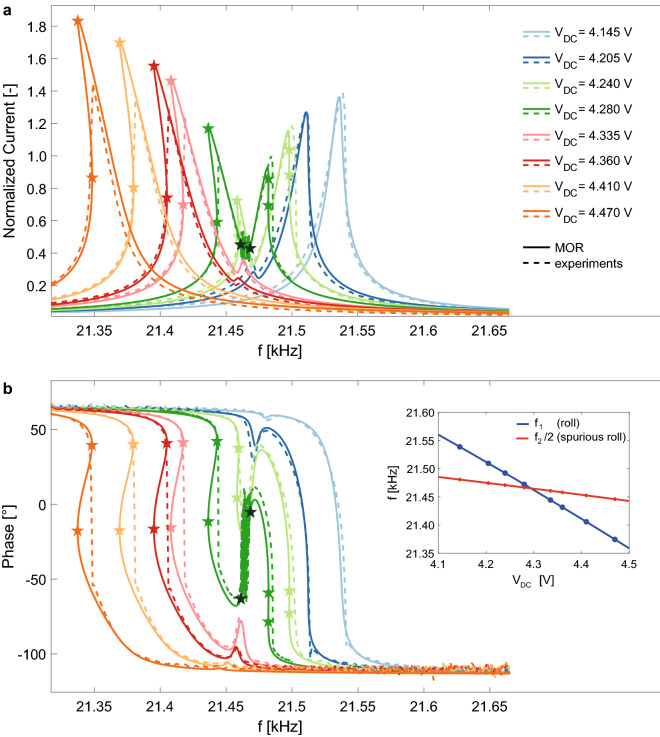
Frequency responses of the MEMS gyroscope test-structure for V_AC_ = 3.16 mV and different levels of V_DC_ in terms of (**a**) amplitude and (**b**) phase. Numerical predictions are reported in continuous lines while experiments are in dotted lines. Stars of the same color of the curves mark the Saddle–Node bifurcations estimated through the ROM model, while black stars mark the Neimark–Sacker bifurcations that delimit the quasi-periodic region of the curves at V_DC_ = 4.28 V. The resonant frequency of the roll mode (*f*_1_) and half of the resonant frequency of the spurious roll mode (*f*_2_/2) are reported in the inset for different V_DC_.

Additional experimental curves measured for a V_AC_ = 3.16 mV and different levels of V_DC_ are reported in dashed-lines in Fig. 3 together with corresponding numerical predictions. Only experimental upward frequency sweeps are reported for the sake of clarity and all the curves, both numerical and experimental, are normalized with the maximum amplitude of the hardening peak of the experimental curve obtained for V_DC_ = 4.28 V and V_AC_ = 3.16 mV. This value corresponds to an out-of-plane maximum displacement of the proof masses of 71 nm. The maximum displacement experienced by the proof masses in this experimental campaign is then in the order of a couple of hundreds of nanometers (i.e. orange curve in Fig. 3a), which is fully compatible with a stable operation of the device far from pull-in instabilities (the gap between the masses and the underlying electrodes is of 1.2 µm) and with the assumption of moderate transformations required by the proposed ICE method. In the inset of Fig. 3b, the evolution of the resonant frequency of the roll mode for different V_DC_ is reported and compared with half the resonant frequency of the spurious roll mode, highlighting the strong link between the nonlinear dynamic behavior of the structure under study and the ratio between the resonant frequencies of the two coupled modes.

A good agreement in terms of amplitude and phase is found for all the DC-voltage levels, thus proving the predictive ability of the simulation tool. It should be recalled that experiments are run in frequency control and frequency upward sweep. As a consequence they cannot follow unstable branches and display jumps in proximity of the Saddle–Node bifurcations predicted by the MOR (see Fig. 2a of Supplementary Information). It is worth noting that the proposed ROM is able to catch the nonlinear dynamic response of the MEMS gyroscope test structure under different actuating conditions without any need of ad-hoc calibration of the coefficients. This make this simulation approach extremily versatile and general.

## Discussion

The proposed ROM based on implicit static condensation is able to accurately and ab-initio reproduce the complex nonlinear dynamics of a MEMS gyroscope test-structure undergoing 1:2 internal resonance including bifurcations of the periodic response. The obtained two degrees of freedom model accounts for the multi-physics nature of the problem and does not require any calibration of the parameters: nonlinear coefficients come indeed exclusively from numerical simulations and can be estimated without the need of experimental data. Moreover, thanks to the reduction of the number of degrees of freedom of the system, simulations run almost real-time and are thus very helpful for design purposes and experimental data post-processing.

This technique represents, to the authors’ best knowledge, the first tool able to estimate a priori and in real-time the nonlinear dynamics of a complex multiphysics system like a MEMS gyroscope test-structure under different actuation conditions.

MEMS designers and the MEMS industry in general, will strongly benefit of such tool since it will simplify the understanding of experimental data and the design process of complex nonlinear MEMS devices.

## Methods

### Implicit static condensation

The implicit static condensation is based on the assumption that it is possible to describe the steady state non-linear oscillation of a resonator as a combination of few master modes (MM). The dynamics of the ROM is described by a stress manifold obtained by implicitely condensing the effects of higher order modes that locally modify the internal forces and thus the global stiffness of the system^42^. For the case under study where two modes interact through the internal resonance, this method allows to formulate a ROM where the active degrees of freedom are the modal coordinates $q_{i}$ of the roll and the spurious roll modes associated to the maximum out-of-plane displacement of the proof masses. Let $\psi_{i} (x)$ denote the displacement field of the *i*-th MM, mass normalized, the non-linear elastic force manifold is evaluated by statically forcing the structure with suitable body forces *F* which are proportional to $\psi_{1} (x)$ and $\psi_{2} (x)$: $F = \beta_{1} \psi_{1} (x) + \beta_{2} \psi_{2} (x)$. The motivation for this choice, apart from simplicity, is that these loads are a very good approximation of inertia forces occurring during the steady state oscillation. Once the body forces are defined, a series of static non-linear analyses are run spanning the $(\beta_{1} ,\;\beta_{2} )$ space. The range of the load-multipliers $(\beta_{1} ,\;\beta_{2} )$ is prescribed so as to cover the expected displacements of the structure, e.g. maximum out-of-plane displacements allowed by the gap between the proof masses and the underlying substrate. Let $(q_{1} (\beta_{1} ,\;\beta_{2} )$,$q_{2} (\beta_{1} ,\;\beta_{2} ))$ denote the solution for a given $(\beta_{1} ,\;\beta_{2} )$, we invert such relations and we obtain the terms $(\beta_{1} (q_{1} ,\;q_{2} )$,$\beta_{2} (q_{1} ,\;q_{2} ))$ of Eqs. (1) and (2).

A similar procedure is adopted to determine the electrostatic nonlinear manifold of the ROM. This represents a quasi-static approach which assumes that the dynamics of electromagnetic forces is much faster than the frequency of oscillation of the resonators, which is verified in the case of the MEMS under consideration. We then suppose that the gyroscope vibrates according to a combination of the two main modes, i.e. roll and spurious roll modes, and we update the coordinates of the conductor surfaces, i.e. surfaces of the proof masses of the gyroscope test-structure that face the underlying electrodes employed for actuation/readout, as $x{ } + \psi_{1} q_{1} + \psi_{2} q_{2}$, being $x$ the initial position of the conductor surfaces. The map of the charge surface density $\sigma (x,\;q_{1} ,\;q_{2} )$ caused by the interaction between the conductor surfaces with the underlying electrodes, is then computed as a function of $(q_{1} ,q_{2} ){ }$ through integral equations accelerated with fast multipole methods^59^. Once the charge surface density is available, the nonlinear load participation factor is computed as:

3 $$F_{{e_{i} }} = \int_{S} {\frac{{\sigma^{2} }}{2}\psi_{{n_{i} }} dS \approx \epsilon_{0} \check{F}_{{e1_{i} }} V_{DC}^{2} ,}$$

where $\psi_{{n_{i} }} = \psi_{i} \cdot n$ is the projection of the modal shape function $\psi_{i}$ along the outward unit normal vector on the conductor surface and *S* is the surface portion of the proof masses that faces the underlying electrodes. An analogous procedure^55^ allows us to determine the nonlinear amplitude of the forcing term $\check{F}_{{e2_{1} }} (q_{1} ,\;q_{2} )$ in Eqs. (1) and (2).

### Experimental set-up

**Figure 4:**
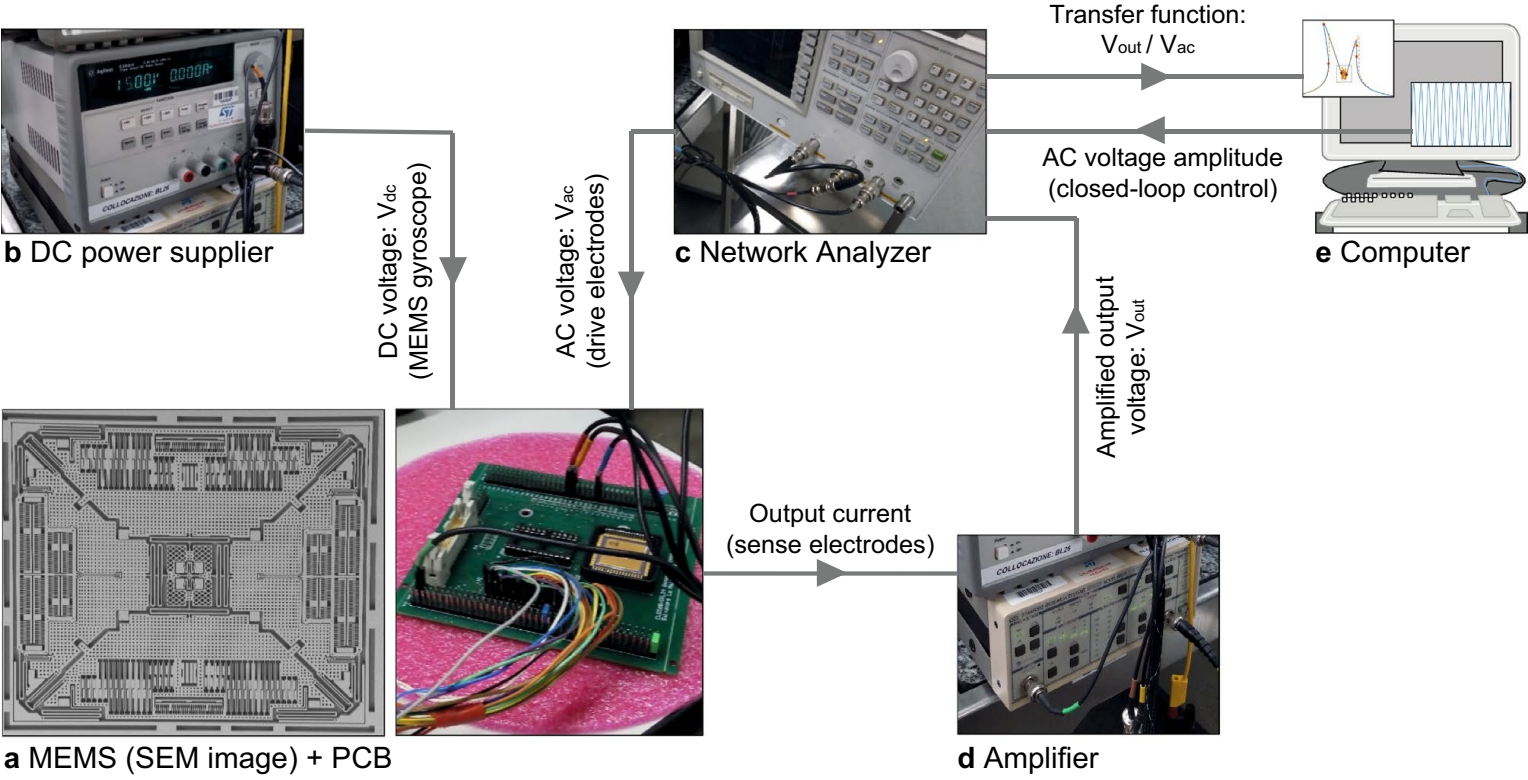
Set-up employed to measure experimental frequency responses.

The MEMS is bonded to a ceramic carrier and then connected to a Plastic Circuit Board (PCB) as shown in Fig. 4a. Electrostatic actuation of the roll mode is provided through two power suppliers (Fig. 4b,c): the Agilent E3631A provides the DC voltage while AC signal is generated through the Agilent AG4395A. The output current measured on the electrodes on the substrate is amplified through the Signal amplifier SRS model SR570 (Fig. 4d) and read in the frequency domain through the Agilent AG4395A (Fig. 4c). A LabView script (Fig. 4e) acquires the output and corrects the AC signal to guarantee a close-loop control of the circuit.

## Supplementary Information

Supplementary Information.
