# Supplementary material for: Reduced order modelling and experimental validation of a MEMS gyroscope test-structure exhibiting 1:2 internal resonance
Source: Sci Rep. 2021 Aug 12;11:16390. doi: 10.1038/s41598-021-95793-y (PMC8361070; doi:10.1038/s41598-021-95793-y)
Supplement: Supplementary file 1 — Supplementary Information. [file 41598_2021_95793_MOESM1_ESM.pdf]

Reduced order modelling and experimental validation of a MEMS gyroscope test-structure exhibiting 1:2 internal resonance

Giorgio Gobat[1], Valentina Zega[1,*], Patrick Fedeli[2], Luca Guerinoni[2], Cyril Touzé[3], Attilio Frangi[1]

[1] Politecnico di Milano, Civil and Environmental Engineering, Milano, Italy

[2] Analog and MEMS group, STMicroelectronics, Cornaredo, Italy

[3] IMSIA, ENSTA Paris, Institut Polytechnique de Paris, Palaiseau, France

*Corresponding Author:

valentina.zega@polimi.it

+390223994213

Supplementary Information

1. MEMS quad-mass gyroscope test structure geometry

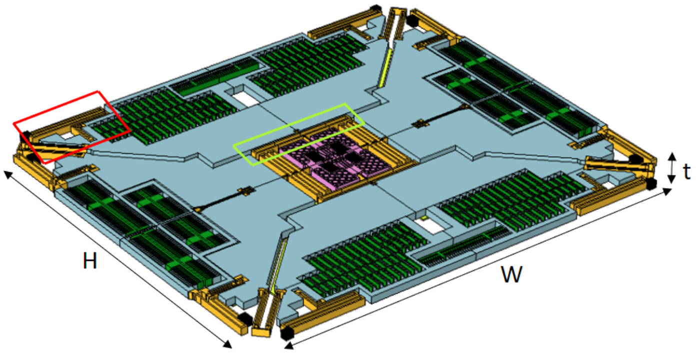

H	1.3 mm
W	1.5 mm
t	24 μm
L	254 μm
h	103 μm
s	4.8 μm
L1	353 μm
s1	4.8 μm

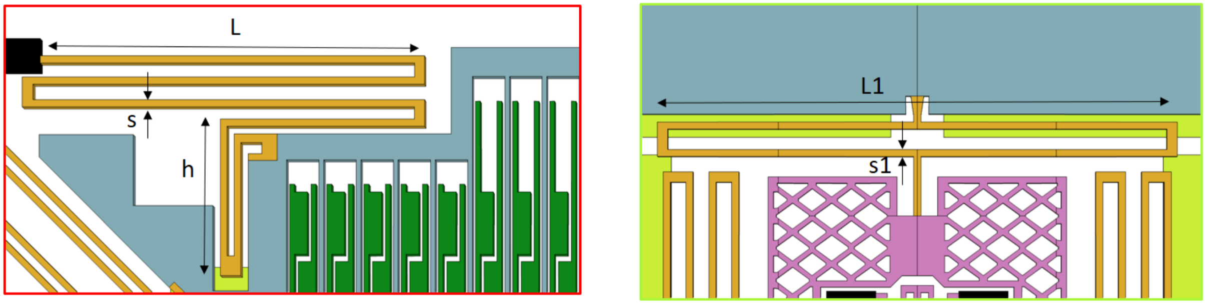

Figure 1: Schematic view of the MEMS test structure. Two close-up views and geometric dimensions are also reported for the sake of clarity.

2. Implicit Condensation Method: nonlinear mechanical and electrostatic forces

In this work, the nonlinear mechanical and electrostatic forces (eqs. (1)-(2) of the paper) computed through the Implicit Condensation Method are interpolated with a complete third order polynomial of the general form:

$$P(q_1, q_2) = c_0 + c_1 q_1 + c_2 q_2 + c_3 q_1^2 + c_4 q_1 q_2 + c_5 q_2^2 + c_6 q_1^3 + c_7 q_1^2 q_2 + c_8 q_1 q_2^2 + c_9 q_2^3.$$

The coefficients utilized for the different contributions are reported in Table 1. It is worth noting that such coefficients do not depend on the V_{DC} and V_{AC} values and are thus able to describe the nonlinear dynamics of the MEMS gyroscope test-structure under different actuating conditions.

	$\boldsymbol{\beta_1(q_1,q_2)}$ [μN]	$\boldsymbol{\beta_2(q_1,q_2)}$ [μN]	$\boldsymbol{\breve{F}_{e1_1}(q_1,q_2)}$ [μN μm/V² pF]	$\boldsymbol{\breve{F}_{e1_2}(q_1,q_2)}$ [μN μm/V² pF]	$\boldsymbol{\breve{F}_{e2_1}(q_1,q_2)}$ [μN μm/V² pF]
$\boldsymbol{c_0}$	3.67 10^{-6}	-2.24 10^{-6}	-0.08555	-284.389	-476.658
$\boldsymbol{c_1}$	0.020024	-2.38 10^{-6}	11.23799	0.087044	5.565844
$\boldsymbol{c_2}$	-2.33 10^{-6}	0.074312	0.087168	9.511499	4.334514
$\boldsymbol{c_3}$	5.19 10^{-11}	-1.75 10^{-8}	-2.68 10^{-5}	-0.071	-0.04597
$\boldsymbol{c_4}$	-1.49 10^{-8}	1.41 10^{-8}	-0.1424	-3.65 10^{-5}	-0.07233
$\boldsymbol{c_5}$	-5.80 10^{-9}	1.14 10^{-9}	-2.12 10^{-5}	-0.03227	-0.02853
$\boldsymbol{c_6}$	-3.05 10^{-11}	-1.69 10^{-12}	0.000826	7.66 10^{-7}	0.000413
$\boldsymbol{c_7}$	-3.80 10^{-12}	-2.83 10^{-10}	1.22 10^{-6}	0.001439	0.000899
$\boldsymbol{c_8}$	-4.01 10^{-10}	-5.49 10^{-12}	0.001488	9.37 10^{-7}	0.000734
$\boldsymbol{c_9}$	-9.80 10^{-12}	-2.03 10^{-9}	4.41 10^{-7}	0.000497	0.000263

Table 1: Coefficients of the polynomial expansion of the mechanical and electrostatic nonlinear forces obtained through the Implicit Condensation Method.

3. Nonlinear frequency response in presence of 1:2 internal resonance

In the presence of a 1:2 internal resonance, the frequency response of each of the two coupled modes takes the peculiar shape reported in Fig.2. As the two peaks of the frequency response are strongly nonlinear, i.e. one hardening and one softening, Saddle-Node bifurcations (red stars in Fig.2) are also present to delimit their unstable branches (red path in Fig.2). Unstable branches cannot be followed experimentally using a standard set-up and, as a consequence, jumps are expected to occur in the proximity of Saddle-Node bifurcations.

The experimental frequency response will be then strongly dependent on the sweep direction as shown by arrows in Figs.2(a)-(b).

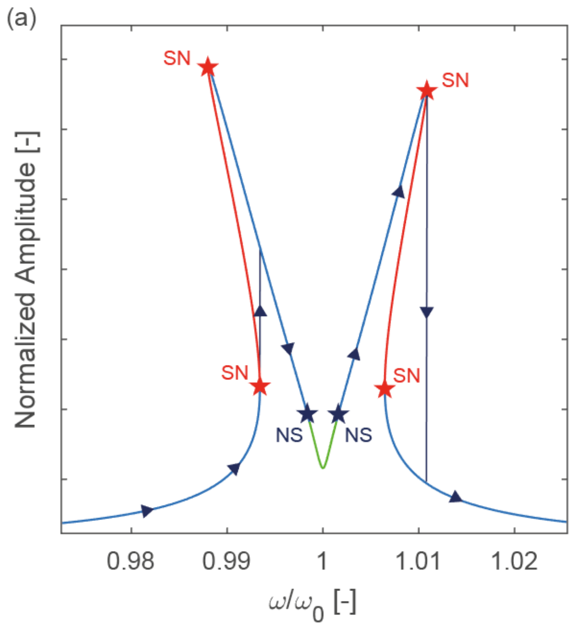

Figure 2: Nonlinear frequency response in presence of a 1:2 internal resonance. (a) Upward and (b) downward frequency sweeps.

It is worth mentioning that a variable-phase feedback loop on the resonator would allow to track the response beyond the critical bifurcation regime[1] but the implementation of such control circuit is outside the scope of the present work.

Finally, Neimark-Sacker bifurcations (dark blue stars) also appear, thus delimiting the quasi-periodic region (green path in Fig.2) where the steady-state periodic response is no longer stable and bifurcates into a quasi-periodic response. This latter is no longer characterized by a single frequency, given by the external excitation, but also by an incommensurate smaller frequency that modulates the response[2]. Moreover, within this region, the quasi-periodic regime can further bifurcate into a nearly chaotic response that is difficult to predict with the available tools. Neimark-Sacker bifurcations have been predicted numerically as illustrated in the body of the paper.

4. Simplified analytical model

In order to further validate the numerical results obtained from the ROM with specific reference to the prediction of the quasi periodic region, we discuss an analytical model obtained starting from the normal form theory. Considering nonlinear terms up to the quadratic order for the sake of simplicity, the normal form for the 1:2 internal resonance reads[2]:

$$
\begin{gathered}
\ddot{q}_1 + 2\mu_1\dot{q}_1 + (\omega_{01}^2 - \breve{\boldsymbol{F}}_{\boldsymbol{e1_1c_1}}\epsilon_0 V_{DC}^2)\delta_{1c_1}q_1 + \left(\boldsymbol{\beta}_{\boldsymbol{1},\boldsymbol{c_4}} - \breve{\boldsymbol{F}}_{\boldsymbol{e1_1c_4}}\epsilon_0 V_{DC}^2\right)q_1 q_2 = 2\,\breve{\boldsymbol{F}}_{\boldsymbol{e2_1c_0}}\epsilon_0 V_{DC} V_{AC}\cos\omega t \\
\ddot{q}_2 + 2\mu_2\dot{q}_2 + (\omega_{02}^2 - \breve{\boldsymbol{F}}_{\boldsymbol{e1_2c_1}}\epsilon_0 V_{DC}^2)\delta_{2c_1}q_2 + \left(\boldsymbol{\beta}_{\boldsymbol{2},\boldsymbol{c_3}} - \breve{\boldsymbol{F}}_{\boldsymbol{e1_2c_3}}\epsilon_0 V_{DC}^2\right)q_1^2 = 0,
\end{gathered}
\tag{1}
$$

where q_i are the modal coordinates, $2\mu_i$ the damping coefficients, ω the external forcing pulsation and δ_{ic_1} are almost unitary correction factors used to force a perfect match of the eigenfrequencies of the model with the measured ones. The other symbols have the same meaning used in the Implicit Condensation approach proposed in the paper.

Coefficients of the analytical model (1) are retrieved from the proposed numerical Implicit Condensation Method (see Table 1 of Supplementary Information) and summarized in Table 2.

As shown in a recent contribution[2], starting from the normal form (1), it is possible to estimate in a closed form both the frequency response curve of the two-dofs system and the Neimark-Sacker boundary curve, i.e. locus of the bifurcation points in the parameters space.

A comparison between the frequency response curve obtained through the full ROM studied in the paper and the simplified analytical model is shown in Fig.3 in terms of amplitude of the modal coordinate of the driven roll mode for a V_{AC}=3.16 mV and a V_{DC}=4.28 V. Red and black stars denote Saddle-Node and Neimark-Sacker bifurcations identified through the numerical model, while the green dashed line represents the analytical Neimark-Sacker boundary curve estimated from eq. (1) through the Multiple Scales Method[2].

Parameter	Value	Units
$\boldsymbol{\omega_{01}^2}$	0.020024	μN/μm
$\boldsymbol{\omega_{02}^2}$	0.074312	μN/μm
$\breve{\boldsymbol{F}}_{\boldsymbol{e1_1c_1}}$	11.23799	μN/ μm V^2 pF
$\breve{\boldsymbol{F}}_{\boldsymbol{e1_2c_1}}$	9.511499	μN/ μm V^2 pF
$\breve{\boldsymbol{F}}_{\boldsymbol{e1_1c_4}}$	-0.1424	μN/ μm^2 V^2 pF
$\breve{\boldsymbol{F}}_{\boldsymbol{e1_2c_3}}$	-0.071	μN/ μm^2 V^2 pF
$\boldsymbol{\beta_{1,c_4}}$	-1.49 10^{-8}	μN/μm^2
$\boldsymbol{\beta_{2,c_3}}$	-1.75 10^{-8}	μN/μm^2
$\breve{\boldsymbol{F}}_{\boldsymbol{e2_1}c_0}$	-476.658	μN/V^2 pF
$\boldsymbol{\delta_{1c_1}}$	0.99948	-
$\boldsymbol{\delta_{2c_1}}$	0.99994	-
$\boldsymbol{\mu_1}$	2.948 10^{-5}	μN μs/μm
$\boldsymbol{\mu_2}$	3.916 10^{-5}	μN μs /μm

Table 2: Coefficients of the simplified analytical model.

A very good agreement between the two models is found in the quasi-periodic region, while small discrepancies are evident at high amplitudes. This is justified by the simplifying hypotheses adopted in the analytical model that does not take into account nonlinearities of higher orders, which are known to play a quantitative role at large amplitudes.

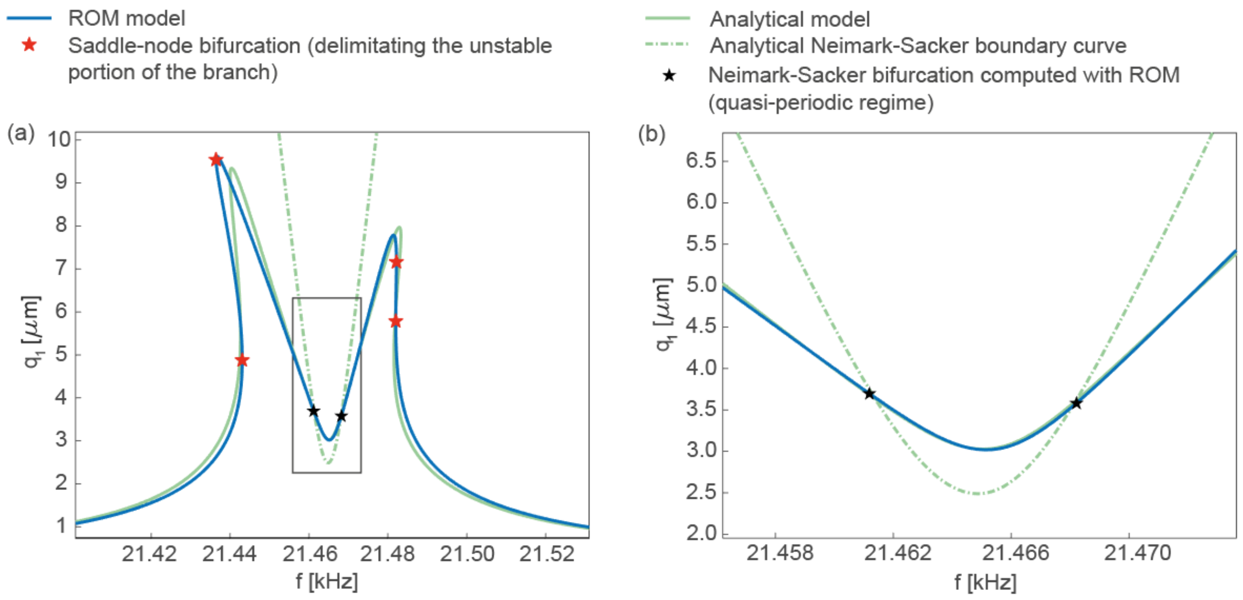

Figure 3: (a) Frequency response of the MEMS gyroscope test-structure for a V_{AC} = 3.16 mV and a V_{DC} =4.28 V. Comparison between the full ROM model proposed in the paper and the simplified analytical model. (b) Close-up view of the quasi-periodic region of the frequency response.

References

1. H. K. Lee et al. Stable operation of MEMS oscillators far above the critical vibration amplitude in the nonlinear regime, *J. Microelectromech. Syst*. **20**(6) 1228-1230 (2011).
2. G. Gobat et al Backbone Curves, Neimark-Sacker Boundaries and Appearance of Quasi-Periodicity in Nonlinear Oscillators: Application to 1:2 Internal Resonance and Frequency Combs in MEMS *Meccanica* (2021) doi: 10.1007/s11012-021-01351-1
